# Dynamic Visual Measurement of Driver Eye Movements

**DOI:** 10.3390/s19102217

**Published:** 2019-05-14

**Authors:** Jin Zhang, Ze Yang, Huaxia Deng, Huan Yu, Mengchao Ma, Xiang Zhong

**Affiliations:** School of Instrument Science and Opto-electronics Engineering, Hefei University of Technology, No. 193 Tunxi Road, Hefei 230009, China; zhangjin@hfut.edu.cn (J.Z.); ze.yang@mail.hfut.edu.cn (Z.Y.); 18225835318@163.com (H.Y.); mmchao@hfut.edu.cn (M.M.); zhx0325@hfut.edu.cn (X.Z.)

**Keywords:** visual fatigue, construction of three-dimensional trajectories, human eye tracking, dynamic visual measurement

## Abstract

Vibrations often cause visual fatigue for drivers, and measuring the relative motion between the driver and the display is important for evaluating this visual fatigue. This paper proposes a non-contact videometric measurement method for studying the three-dimensional trajectories of the driver’s eyes based on stereo vision. The feasibility of this method is demonstrated by dynamic calibration. A high-speed dual-camera image acquisition system is used to obtain high-definition images of the face, and the relative trajectories between the eyes and the display are obtained by a set of robust algorithms. The trajectories of the eyes in three-dimensional space are then reconstructed during the vehicle driving process. This new approach provides three-dimensional information and is effective for assessing how vibration affects human visual performance.

## 1. Introduction

The vibration of vehicles often causes visual and physical fatigues for the passengers, but drivers also encounter these problems. Visual fatigue is one of the types of fatigue driving and seriously affects the driver reaction time. Traffic accidents caused by visual fatigue and drowsiness cause considerable economic losses [1,2]. For armored vehicles and tanks, displays are essential for monitoring the surroundings. Military vehicles are routinely equipped with direct-view optical systems, cameras, laser range finders, and thermal imagers to observe the surrounding environment and destroy targets. However, it is well known that vibrations increase the difficult of reading digital displays, and misjudgments and improper operation under vibration conditions can lead to unsafe incidents.

Regarding how vibration affects human visual performance, Moseley et al. [3] studied how (i) display vibration, (ii) whole-body vibration, and (iii) the simultaneous vibration of both the display and the observer affect visual performance. Lewis et al. [4,5] studied how the vibration frequency, viewing distance, and seating conditions affect the reading of numeric displays. Lin et al. [6] found that using larger fonts and fewer characters can reduce the effects of vibration on reading performance and visual fatigue when identifying numeric characters. Because vehicle displays can be considered to be stationary, it is mainly whole-body vibration that affects visual performance and causes visual fatigue. Under the condition that the vibration frequency is lower than 1 Hz, the observer can track targets, but soon experiences fatigue, while the observer cannot track targets if the frequency is higher than 2 Hz [6]. Horng et al. [7] found that the vision and stereopsis of participants declined considerably when the acceleration exceeded 0.1 g. Furthermore, as listed in Table 1, standard ISO 2631-1:1997 [8] defines the relationship between acceleration and bodily sensation. Consequently, there is an urgent need for a method of measuring vibration and evaluating its effects on the visual fatigue of drivers.

Driver fatigue is usually assessed using non-contact measurements because contact measurements are intrusive. The vibration measurement device attached to the driver seat can measure the vibration of the seat, but it cannot determine whether the driver is fatigued or not. Driver fatigue is affected by many aspects. By directly detecting the driver’s eye, the lever of fatigue can be determined. In driver-assistance systems, the most widely-used type of non-contact measurement is visual measurement [9]. The driver’s eye state and head pose can be estimated using a monocular camera as a strong indicator of her/his mental state and level of drowsiness [1,2,9,10,11,12]. Alioua et al. [10] estimated the discrete head pose to predict the level of driver attention. Jimenez-Pinto et al. [1] obtained the shape of eyes and mouth to predict whether the driver was yawning or blinking. Ji et al. [9] estimated the pupil position, gaze direction, and facial orientation to monitor driver vigilance; however, they estimated the head pose and eye position roughly in a two-dimensional plane and could not provide an accurate trajectory in three-dimensional (3D) space. Except for eye state and head pose, eye movement is another criterion for evaluating mental state and visual fatigue. Human eye movements and gaze direction can be estimated by measuring the circularity and centroid of the iris [13,14]. Zhang et al. [14] made a preliminary estimation of eye gaze from the elliptical features of one iris and obtained the vectors describing the translation and rotation of the eyeball; however, they did not investigate the associated head movements. Overall, the aforementioned studies show that visual measurement technology is widely used in the dynamic detection of human eyes.

The aforementioned studies were concerned with dynamic detection, which is different from dynamic tracking. The former estimates the head posture and gaze direction at a given moment only, whereas the latter measures the movement trajectory over a period of time. Although existing methods can perform dynamic detection of the head and eyes, they cannot be used for dynamic tracking. Zhang et al. [15] used sub-pixel interpolation for visual measurement and conducted highly-accurate 3D reconstruction of vibration based on stereo vision. Baqersad et al. [16] measured the dynamic strain on a wind turbine by means of visual measurement, and Yang et al. [17] used a videometric technique to measure large-scale blade deformations. Previously, we used a binocular stereo vision system to identify the unbalanced mass of rotor systems in 3D space [18]. However, although methods already exist for tracking objects, there is an urgent need for a new method for the dynamic tracking of human eyes.

Herein, the focus is on a new eye-tracking method. The motion of the eyes relative to the cameras is measured accurately using a binocular stereo vision system. The precision of 3D measurement in the binocular stereo vision system can reach 0.1 mm, thereby guaranteeing the reconstruction of 3D eye trajectories. The visual measurement method is a non-contact and full-field optical measurement method that can accurately reconstruct the 3D trajectories of objects by using a non-contact measurement device. Consequently, the proposed method could be used in a driver-assistance system to monitor driver fatigue with the aim of avoiding traffic accidents. The rest of this paper is organized as follows. In Section 2, we describe the experimental equipment and algorithm flow. In Section 3, we present a feasibility study of accurate eye tracking based on stereo vision. We present and discuss the results of the present experiments in Section 4 and Section 5, and we present fatigue study in Section 6. Finally, we put forward our conclusions in Section 7.

## 2. Methods

For the stereo video system, the 3D reconstruction accuracy is affected by the intrinsic and external parameters of the cameras, which can be calibrated using a checkerboard [19]. The process of camera calibration is referred to as static calibration. Meanwhile, providing a standard motion trajectory to verify the correctness of the 3D reconstruction is referred to as dynamic calibration. Herein, the standard motion trajectory was provided by a vibration table to validate the proposed method.

**Figure 1:**
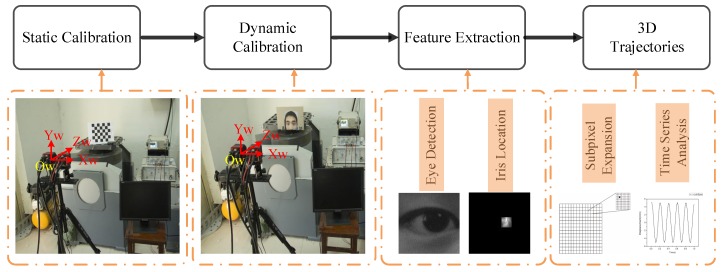
Procedure for reconstructing three-dimensional (3D) eye trajectories.

Figure 1 shows the flowchart of the proposed method. Using the checkerboard for static calibration gives the positions of the two cameras relative to each other. The world coordinate system $O-X_{w}Y_{w}Z_{w}$ was centered on the left-hand camera. The feature points of the eyes were obtained by feature extraction applied to the collected images, whereupon the 3D coordinates and trajectories of the eyes were obtained by means of a reconstruction model and the associated formula.

In the structure of the human eye, the most obvious features are the outline and center of the iris. However, with the existing algorithms, these cannot be extracted directly and precisely from a picture that contains a complete human face [20,21]. It is therefore necessary to divide the positioning process into several parts. First, the separation algorithm for the eyes comprised the template matching, a method based on a skin-color model, and a method based on machine learning [22,23,24,25]. When reconstructing the 3D coordinates, the first step to recognize the eyes, for which the robust Haar cascade classifier separation algorithm [25,26], was used to perform eye location and extraction within the images. Second, the Daugman circular integro-differential operator [27,28,29] was utilized to extract the precise pixel coordinates of the center of the iris in the eye region of interest (ROI) obtained in the first step. Compared with other algorithms for iris identification, the main characteristic of the present algorithm is its superior stability. Finally, because of the limitations of the Daugman operator, the Taylor interpolation algorithm [15] was used to further obtain the sub-pixel coordinates.

The Haar cascade classifier is a cascade classifier that is based on Haar-like features, the main ones being edge features, line features, and center-surround features [30,31]. The file haarcascade_eye.xml included in OpenCV is used for eye location and extraction. Compared with other similar files, the present one is more suitable for extracting smaller areas to narrow down the calculation range of the next step. When human eyes are searched for within subsequent images, the new ROI is selected based on the eye positions in the previous images. This method ensures accuracy, greatly reduces the search time, and improves the real-time performance of the algorithm.

The boundary of the iris is defined as two circles: the boundary between the pupil and the iris is the inner circle, and the boundary between the iris and the sclera is the outer circle [29]. People of different ethnicities have different iris colors; for example, brown eyes contain more melanin, but blue eyes contain less. Consequently, some people have low contrast between pupil and iris, which makes it difficult to identify the inner circle. However, the center of the outer circle is very close to that of the inner circle, so the center of the outer circle is extracted as that of the iris in the method proposed herein.

**Figure 2:**
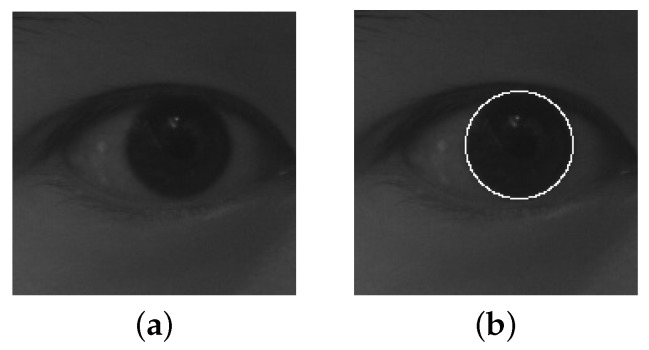
Eye-containing image processed using Daugman operator: (**a**) original image; (**b**) processed image.

The Daugman operator is utilized to calculate the circle with the maximum pixel-value difference between the inner and outer iris edges with single-pixel precision [27,28]. Unlike the circular Hough transform, the Daugman operator gives one final result and does not require a threshold to be set. Moreover, the Daugman operator is unaffected by the average light intensity, making it more suitable for automated processing. The Daugman operator is defined as:

(1) $$\max_{\left(r,x_{0},y_{0}\right)}G_{\sigma }\left(r\right)*\frac{\partial }{\partial r}\oint_{r,x_{0},y_{0}}\frac{I(x,y)}{2\pi r}ds,$$

where I(x,y) is the image and $G_{\sigma }\left(r\right)$ is a Gaussian smoothing function. The loop integral is calculated by taking $\left(x_{0},y_{0}\right)$ as the center point and *r* as the radius. Eye-containing image is processed using Daugman operator, which is shown in Figure 2. The Daugman operator takes each pixel in the image as a preselected center of the iris and then varies the radius and center position of the circular contour. Consequently, it terminates with single-pixel precision.

However, dynamic measurement requires extremely high accuracy to extract feature points. Here, the feature point, namely the center of the eye, corresponds to many pixels in the imaging plane, meaning that sub-pixel technology must be adopted. Previously, we used a Taylor expansion for dynamic measurement [15]. The Taylor expansion of the scale-space is calculated as:

(2) $$D\left(x\right)=D+\frac{\partial D^{T}}{\partial x}x+\frac{1}{2}x^{T}\frac{\partial^{2}D}{\partial x^{2}}x,$$

where $\hat{x}$ is an offset between the extreme position and the picture element position and *D* and its derivative are calculated from the feature point and its neighbors. We obtain $\hat{x}$ from:

(3) $$\hat{x}=-\frac{\partial^{2}D^{-1}}{\partial x^{2}}\frac{\partial D}{\partial x}.$$

If $\hat{x}$ is greater than 0.5, then the extreme position is around other pixels. Once $\hat{x}$ is less than 0.5, the extreme position can be determined with sub-pixel precision.

## 3. Dynamic Calibration

To verify that stereo vision can reconstruct accurately the 3D trajectories of human eyes, dynamic calibration must be adopted. The dynamic calibration process involves (i) camera calibration, (ii) eye ROI extraction, (iii) iris localization, (iv) reconstruction of 3D trajectories, and (v) error analysis. A facial image was printed as a photograph and attached to the vibration table, the latter being used to provide a standard sinusoidal motion. The motion frequency and amplitude were set to 5 Hz and 10 mm, respectively, while the camera sampling frequency was 128 Hz.

**Figure 3:**
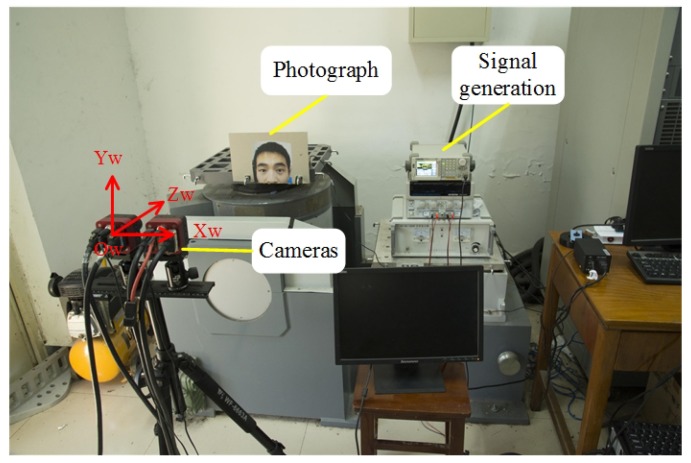
Experimental setup for the dynamic calibration.

The experimental setup is shown in Figure 3. The vibration table, which was supplied by the Suzhou Testing Instrument Company, provided a standard sinusoidal motion in the vertical direction. The image acquisition system contained two high-speed cameras, two image acquisition cards, a signal generator, and a computer. The cameras were mounted on a tripod and placed in front of the vibration table so that the optical axis of the left-hand camera was perpendicular to the photograph. The signal generator produced a trigger signal to synchronize the two cameras. The laser displacement sensor that was used to detect the actual motion of the vibration table was supplied by the Micro-Epsilon Measurement Company.

**Figure 4:**
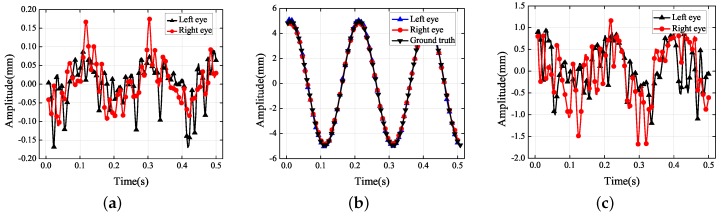
Experimental results for the amplitude in each translational direction: (**a**) *X* axis; (**b**) *Y* axis; (**c**) *Z* axis.

The dots in Figure 4 represent the final 3D reconstruction results. The average value of the data was set to zero for ease of analysis. The results in Figure 4b were fitted with a sinusoidal curve that coincided with the standard motion. Table 2 contains the more-accurate analysis data of Figure 4b. The ground truth representing the vibration information was measured using the laser displacement sensor, whose measurement precision was 0.001 mm.

According to Figure 4b, the amplitudes of the left and right eyes in the vertical direction were respectively 5.032 and 4.844 mm, which were both very close to the ground truth of 4.932 mm; the error was approximately 0.1 mm. The vibration had small non-zero components along the *X* and *Z* axes of the world coordinate system because it was difficult to make the optical axis of the left-hand camera strictly perpendicular to the photograph. In Figure 4c, there is an amplitude of approximately 1 mm along the *Z* axis of the world coordinate system. Because it is easier to adjust the misalignment of the *X* axis of the world coordinate system, it can be seen that the amplitude in the *X* direction was more acceptable than that in the *Z* direction. Because of errors in the 3D reconstruction, the trajectories in the *X* and *Z* directions did not coincide with the standard sinusoidal curve. Although the amplitudes in the *X* and *Z* directions were not controlled to be close to zero, they were both within a reasonable range nevertheless.

**Figure 5:**
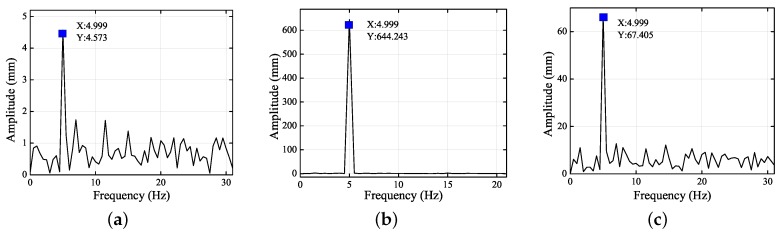
Frequency spectra of the left eye in the (**a**) *X*, (**b**) *Y*, and (**c**) *Z* directions.

**Figure 6:**
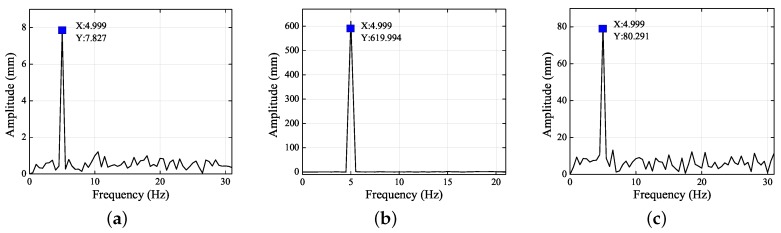
Frequency spectra of the right eye in (**a**) *X*, (**b**) *Y*, and (**c**) *Z* directions.

Figure 5 and Figure 6 show the frequency spectra of the left and right eyes in the *X*, *Y*, and *Z* directions. When the amplitude reached its maximum, the frequencies were all 4.999 Hz. Thus, the standard vibration of 5 Hz was the main factor influencing the motions in the *X*, *Y*, and *Z* directions. As analyzed in the previous paragraph, the standard vibrations affected the motions in the *X*, *Y*, and *Z* directions differently. From Figure 5 and Figure 6, it is clear that the signal-to-noise ratio (SNR) in the *Y* direction was the least, followed by the *Z* direction, and then the *X* direction. The images of the left and right eyes were almost identical in both the time domain and the frequency domain, thereby showing that either eye can correctly reflect the 3D trajectory of the head movement. Consequently, the present experiment demonstrated at length that the 3D eye trajectories can be reconstructed using stereo vision.

**Figure 7:**
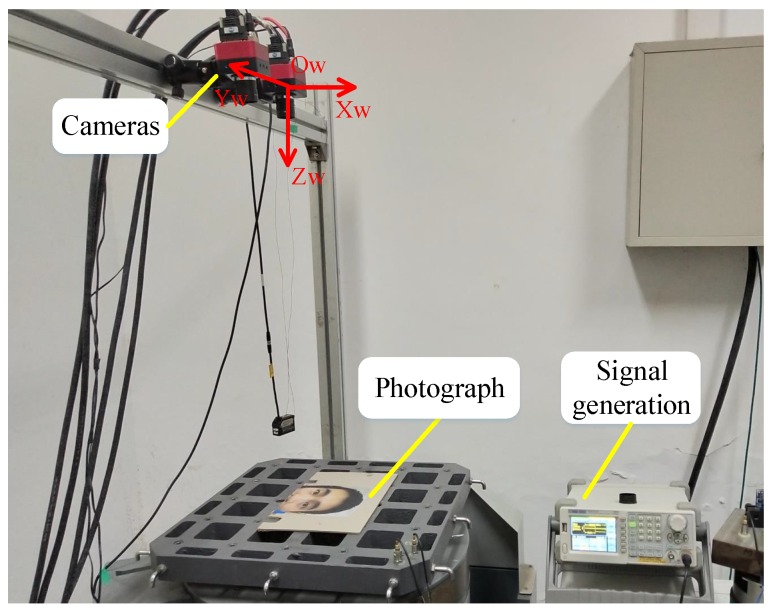
Experimental setup for dynamic calibration (movement in the *Z* axis).

As can be seen from the above experiment, the movement of the human eye in the *Y* axis can be correctly measured. The *X* axis and the *Y* axis were both in the imaging plane of left camera, so their motion laws were consistent. However, the *Z* axis was perpendicular to the imaging plane of the camera. Therefore, it was necessary to verify whether the movement in *Z* axis was close to the true value. Figure 7 shows the experimental setup. The dual camera was fixed on the gantry. The vibration table provided a standard sinusoidal motion, whose motion frequency and amplitude were 5 Hz and 10 mm, respectively.

**Figure 8:**
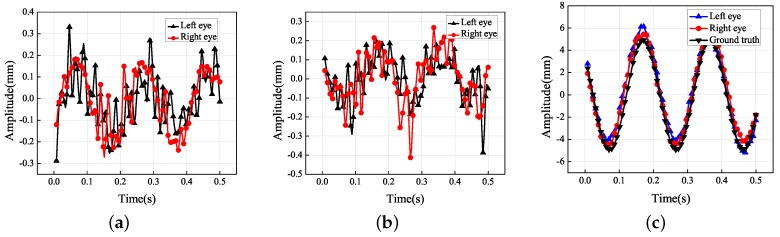
Experimental results for the amplitude in each translational direction: (**a**) *X* axis; (**b**) *Y* axis; (**c**) *Z* axis.

The dots in Figure 8 represent 3D reconstruction results. The results in Figure 8c were fitted with a sinusoidal curve that coincided with the standard motion. Table 3 contains the more accurate analysis data of Figure 8. The amplitudes of the left and right eyes in the *Z* direction were respectively 4.963 and 4.983 mm, which were both very close to the ground truth of 4.909 mm. The detection value (L) was not as good as the value (R) due to the extraction of the feature points, but it was also within an acceptable range.

**Figure 9:**
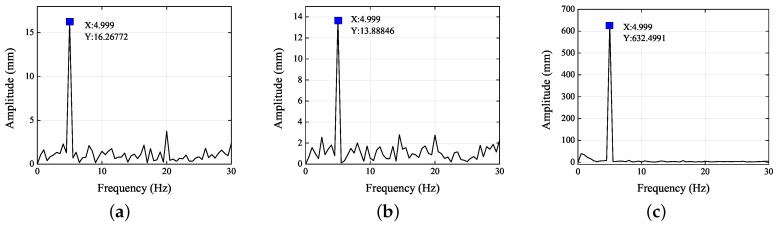
Frequency spectra of left eye in the (**a**) *X*, (**b**) *Y*, and (**c**) *Z* directions.

**Figure 10:**
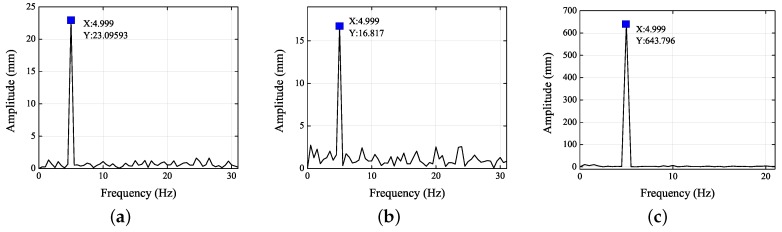
Frequency spectra of right eye in the (**a**) *X*, (**b**) *Y*, and (**c**) *Z* directions.

Figure 9 and Figure 10 show the frequency spectra of the left and right eyes in the *X*, *Y*, and *Z* directions. It is clear that the SNR in the *Z* direction was the least, followed by the *X* direction, and then the *Y* direction.

Consequently, the present experiment demonstrated at length that the 3D eye trajectories can be reconstructed using stereo vision. According to the results of the present experiments, dynamic eye tracking based on stereo vision is an alternative way to measure human motion or vibration. It is a non-contact method that can locate the eyes in less than 0.01 s and can perform accurate 3D spatial reconstruction.

## 4. Experiment and Results

**Figure 11:**
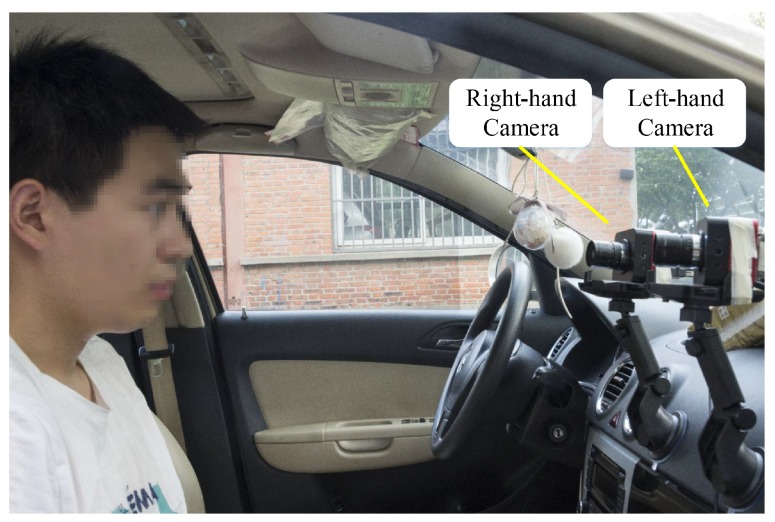
On-board image acquisition system.

To obtain and explore the movement information of human eyes in real life, the experimental device was fitted to a vehicle. The high-speed dual cameras were fixed in front of the front passenger seat as shown in Figure 11. When the car was moving, the cameras measured the motion of the eyes relative to the car. As in the previous experiment, the world coordinate system $O-X_{w}Y_{w}Z_{w}$ was centered on the left-hand camera, and the optical axis of the left-hand camera was as perpendicular as possible to the eyes. The image acquisition frequency was set to 128 H,z and the acquisition time was set to 2 s, whereupon experimental research was conducted on the 3D trajectories of the eyes during the vehicle driving process. A private vehicle was used for tests on flat roads and bumpy roads, while the measured participant did not wear a seat belt and relaxed so that data could be obtained free from interference.

**Figure 12:**
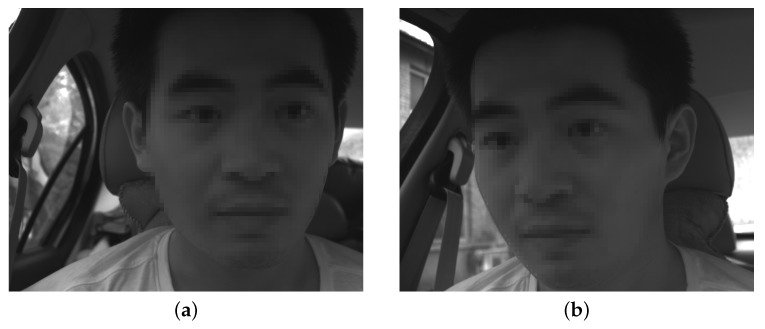
Facial images captured by binocular stereo video system: (**a**) left-hand camera; (**b**) right-hand camera.

The facial images taken by the left-hand and right-hand cameras are shown in Figure 12. Visual measurements were more susceptible to the surrounding environment, and the background of the vehicle was more complicated and the ambient light stronger than in the laboratory environment. Moreover, the distance between the head and the cameras changed constantly while the vehicle was in motion, which sometimes resulted in blurry facial images. The reconstruction accuracy will be affected, and the 3D trajectories will be corrupted by incorrect results unless a stable algorithm is used.

While the vehicle was in motion, the free movement of the eyes and head was recorded by the two high-speed cameras. The projection of each trajectory in the *X*, *Y*, and *Z* directions is respectively plotted. The triangles represent the 3D trajectories of the left eye, and the circles are the corresponding data for the right eye. All results took the center point of the left camera as the origin of the world coordinate system. As long as the head did not rotate excessively, the distances between the left and right eyes in the *X*, *Y*, and *Z* directions would be basically fixed.

**Figure 13:**
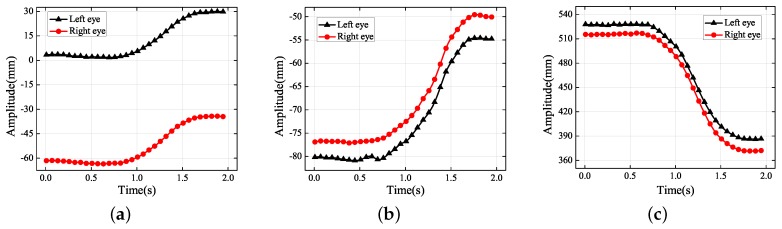
Experimental results obtained during car braking: (**a**) *X* axis; (**b**) *Y* axis; (**c**) *Z* axis.

**Figure 14:**
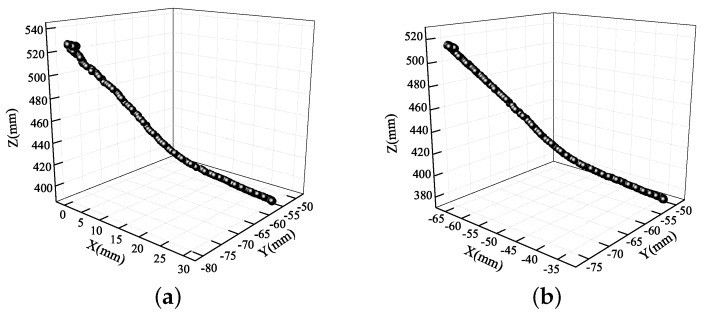
3D eye trajectories during car braking: (**a**) left eye; (**b**) right eye.

First of all, a braking experiment was carried out on a flat road. Braking is the most common action when the road terminates or there are obstacles, but smooth braking and sudden stopping create different travel experiences. Moreover, frequent braking can give rise to physical and visual fatigue, as well. The projections of the trajectories and the 3D trajectories are presented in Figure 13 and Figure 14, respectively. Figure 13 is composed mainly of two straight lines and one oblique line. The two straight lines represent the subject’s body being stationary, while the oblique line indicates that the subject’s body is moving. The displacements in the *X* and *Y* directions were, respectively, 30 mm and 25 mm, which are shown in Figure 13a,b. As indicated in Figure 13c, the eyes moved ever closer to the cameras, and the forward acceleration was lower than 0.03 g according to the definition of the world coordinate system. According to ISO 2631-1:1997, the driver’s vision was unaffected and the driver did not feel anything. Therefore, once the eye acceleration exceeded a certain level, the method proposed in this paper can identify different levels of eye fatigue.

The following 3D trajectories show more detailed trajectories of both eyes. Each point in the 3D scatter diagram corresponds to a position of the eyes at different times. The 3D trajectories of the eyes during car braking are shown in Figure 14. The two trajectories were from top left to bottom right, which means that the head moved forward by approximately 140 mm, right by 30 mm, and upward by 25 mm.

**Figure 15:**
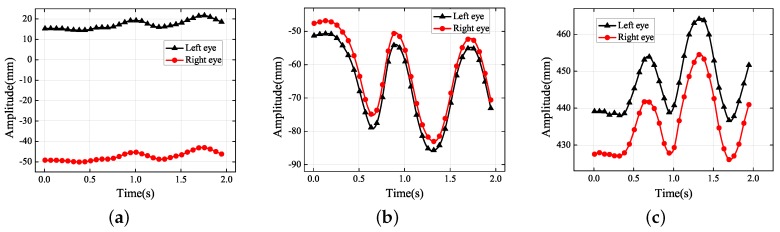
Experimental results obtained during driving over speed bumps: (**a**) *X* axis; (**b**) *Y* axis; (**c**) *Z* axis.

After that, the experiment, i.e., the car went over several speed bumps quickly, was performed. When driving on a bumpy road or going over a speed bump, car vibration is inevitable. To reduce the feeling of shaking involves the design and optimization of the vehicle suspension systems and seats. The projections of trajectory are clearly shown in Figure 15. The frequency of vibration was approximately 1.3 Hz. The amplitude in the *Y* direction was about 30 mm and that in the *Z* direction 15–25 mm, and the lateral displacement was negligible. It would be difficult for the driver to track targets under these circumstances. On city roads, the vibrations generally occur in the *Y* and *Z* directions. However, when a car travels down a country road, roll vibrations of the human body often happen as the car tilts. Vehicles should be designed and adjusted differently for different road surfaces.

**Figure 16:**
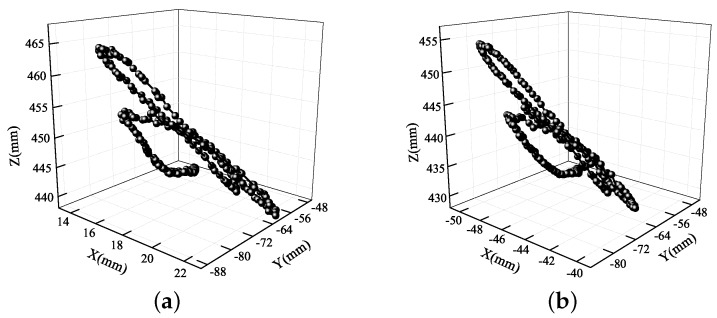
3D eye trajectories during driving over speed bumps: (**a**) left eye; (**b**) right eye.

The two 3D trajectories in Figure 16 are not exactly same, showing that the movement trajectories of the left and right eyes differed in the vibrational environment. This was caused by the slight roll vibrations of the human body. Moreover, the motion trajectory was not a straight line, which cannot be found from the 2D information. By reconstructing the 3D eye trajectories, human vibration had also been measured quantitatively. This is why the shock absorption of vehicles and seats is of great interest: it is difficult to finish a reading task under the condition of high frequency vibration, and visual and physical fatigue are easily caused.

**Figure 17:**
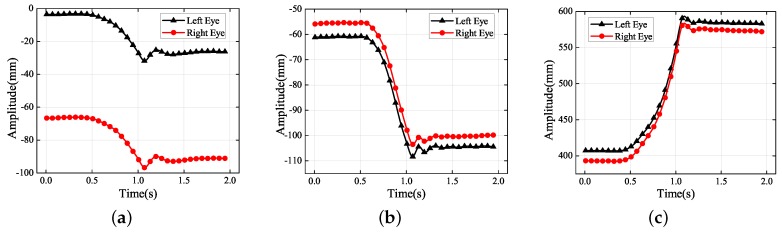
Experimental results obtained during car acceleration: (**a**) *X* axis; (**b**) *Y* axis; (**c**) *Z* axis.

Finally, with the vehicle accelerating, the head movement was recorded. Vehicle acceleration is one of the main indexes of vehicle performance, and it determines transport performance. The results of experiment are shown in Figure 17, which is generally similar to Figure 13. The displacements in the X and Y directions were respectively 30 mm and 45 mm, which are shown in Figure 17a,b. The head moved backward by approximately 200 mm, and the backward acceleration was approximately 0.05 g according to Figure 17c. The three spikes in Figure 17 corresponded to the head hitting the seat back and then bouncing forward. The rebound observed came from a combination of body resistance and head hitting. Under such conditions, the driver’s vision was unaffected, but she/he will feel very mild discomfort.

**Figure 18:**
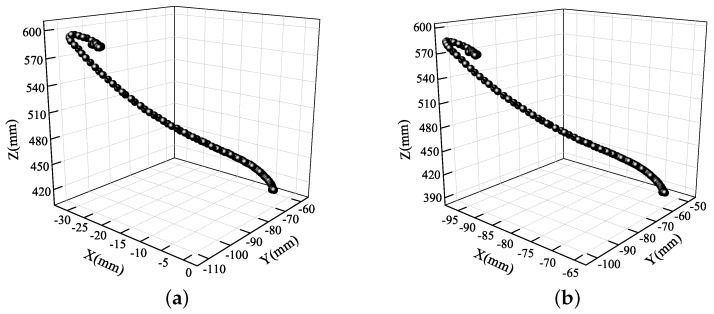
3D eye trajectories during car acceleration: (**a**) left eye; (**b**) right eye.

Although Figure 18 has a shape very similar to that of Figure 14, the eye movement is in the opposite direction. Because the eyes moved away from the cameras at a higher speed, the 3D scatter was not as continuous as it was in Figure 14. After the head touches the seat back, there was a certain degree of rebound. However, the rebound motion was not a simple repeat, but instead changed slightly in the *X*, *Y*, and *Z* directions. Therefore, by studying the movement of the human body during acceleration, the seat can also be better designed and adjusted.

**Figure 19:**
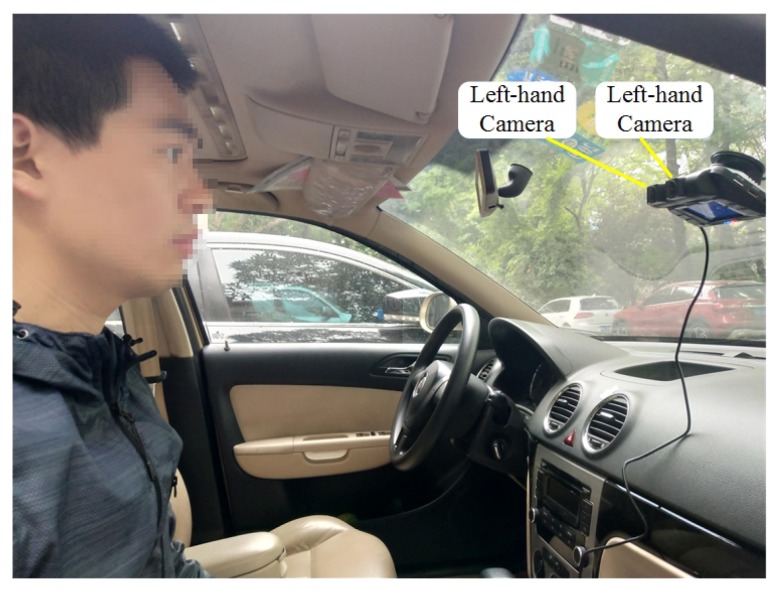
On-board image acquisition system (drive recorder).

**Figure 20:**
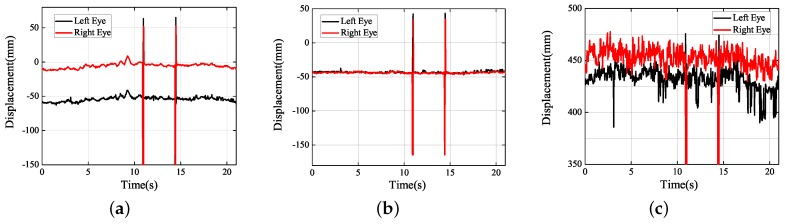
Experimental results obtained (10 km/h): (**a**) *X* axis; (**b**) *Y* axis; (**c**) *Z* axis.

**Figure 21:**
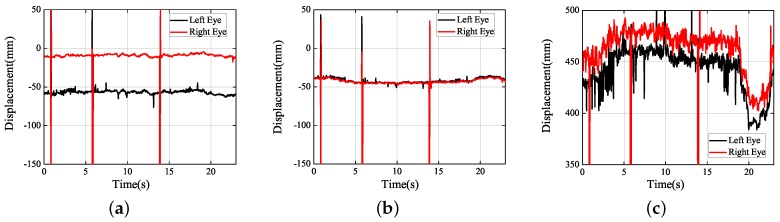
Experimental results obtained (20 km/h): (**a**) *X* axis; (**b**) *Y* axis; (**c**) *Z* axis.

**Figure 22:**
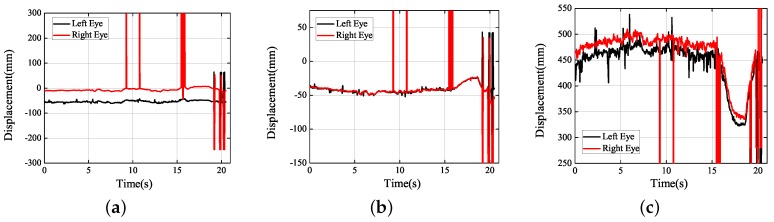
Experimental results obtained (30 km/h): (**a**) *X* axis; (**b**) *Y* axis; (**c**) *Z* axis.

**Figure 23:**
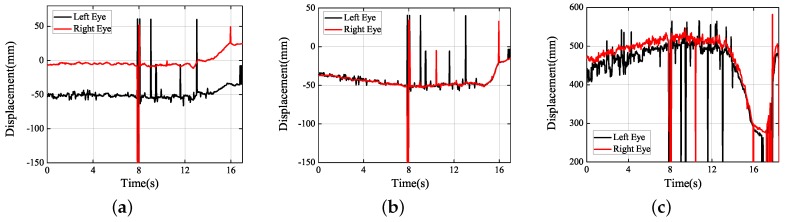
Experimental results obtained (40 km/h): (**a**) *X* axis; (**b**) *Y* axis; (**c**) *Z* axis.

**Figure 24:**
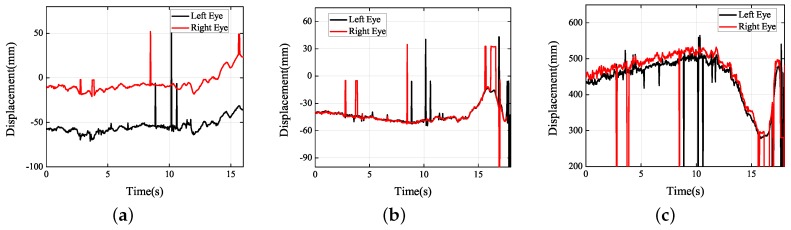
Experimental results obtained (50 km/h): (**a**) *X* axis; (**b**) *Y* axis; (**c**) *Z* axis.

Next, under the real road conditions, the three experiments studied above were carried out for a long time, namely braking experiment, speed bump experiment, and vehicle accelerating experiment. Due to the power supply of the equipment, we replaced the dual high-speed camera with a driving recorder with dual cameras, which was provided by Shenzhen Jiafeng digital communication technology company. The experimental device is shown in Figure 19. The movements of human eye in response to the different speeds were recorded at five different speeds, including 10 km/h, 20 km/h, 30 km/h, 40 km/h, and 50 km/h. Each experiment involved acceleration, constant speed, and braking. The experimental results are shown in Figure 20, Figure 21, Figure 22, Figure 23 and Figure 24. The trajectories of the left and right eyes are indicated by black and red lines, respectively. From these figures, the motion trajectories of human eyes during acceleration and braking (including the backward movement during acceleration and the forward movement during braking), as well as the time taken for acceleration and braking can be seen. The vertical lines in each picture were caused by closed eyes or the wrong calculation of the algorithm. In order to show the real results, these wrong data were not filtered out.

It can be shown from Figure 20 that when the speed was accelerated from 0 km/h–10 km/h and from 10 km/h–0 km/h, no apparent movement was shown in the direction of *X*, *Y*, and *Z*. Therefore, under the slow movement of the vehicle, almost no shaking can be felt, which was caused by the resistance of the body. When the vehicle speed was accelerated to 20 km/h, apparently it is shown from the Figure 21c, at 20 s, that when the vehicle was braked, the human eyes moved forward for 50 mm and stopped at two seconds. Later, the red and black curves apparently returned to 450 mm or so. Owing to the sitting posture being upright, but not leaned forward, when the vehicle was completely stopped, the human body would be automatically recovered to the upright sitting posture.

It can be shown from Figure 22c that when the vehicle started to be braked at 30 km/h, the human eyes moved forward for a larger distance, which reached 100 mm. Seemingly, when the vehicle was completely stopped, the human body would be automatically recovered to the upright sitting posture. However, different from Figure 21c, the time that it took for the human to be recovered to the upright sitting posture was shorter than the one, which was taken for the head to go forward when it braking. At this moment, the thing that the driver’s body was mainly influenced by was the movement process that the human body had recovered to the upright sitting posture.

Human eye’s movement trails have been respectively represented in Figure 23 and Figure 24 at the speeds of 40 km/h and 50 km/h. When it was accelerated to a higher speed, the human eye’s movement became more active in the directions of *X* and *Y*. Not only that, owing to faster accelerated speed when the car was accelerated, the process of the human eyes’ moving back during 0–10 s can be seen. Seemingly, the head’s accelerated speed of moving back was greater than the one of head’s moving forward. At this moment, the human eyes’ accelerated speed could be calculated to be 0.02 and was close to 0.03 g.

By analyzing the 3D information about the human eyes’ movement under five different vehicle speeds, the information about the head movement can be collected as the Table 4. Besides, at the right of each accelerated speed, the body feeling it corresponded to is attached. However, it was found that when driving on the normal road, the acceleration was not large.

It is well known that when a car is passing a speed bump, the driver’s head can move up and down, left and right, and forward and backward. The vehicle we were driving was passing the speed bump respectively at the speeds of 10 km/h, 20 km/h, 30 km/h, and 40 km/h, and the displacement of the driver’s head at the direction of *X* axis, *Y* axis, and *Z* axis is listed in Table 5. It can be shown from Table 5 that when the speed of the vehicle was at about 20 km/h or so, the vibration amplitude of the drivers’ head was smaller and the frequency at the moment was lowest, which was about 1 Hz. When the speed of the vehicle was 30 km/h, the vibration amplitude of the driver’s head was smallest and human body could not feel any apparent shaking. People often think that the lower the speed is, the better it will be when passing the speed bump. However, according to the table, when the car speed was at 10 km/h, the human body’s head was shaken apparently, and the vibration frequency was larger. When the vehicle speed was at 40 km/h, owing to the faster speed, the decline of the vehicle’s speed was more apparent when it was passing the speed bump, then the amplitude of displacement was larger. By considering the vibration amplitude and frequency overall, it can be thought that a better experience can be brought by common private cars at speeds of 20–30 km/h.

**Figure 25:**
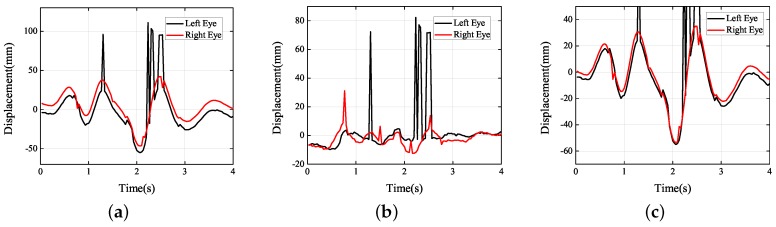
Experimental results obtained: (**a**) *X* axis; (**b**) *Y* axis; (**c**) *Z* axis.

Besides, in order to verify the human body’s roll vibration due to the leaning vehicle body when it passes the speed bump, the experiment of the leaning vehicle body when the car passed the speed bump at a smaller speed was designed in the article. When the vehicle speed was at 10 km/h, the tire of the vehicle at one side passed the speed bump, and the driver’s head was also shaking violently. The experimental results dealt with and collected are shown in Figure 25. In the direction of *Y*, there are apparently crosses on the black line and red line, showing that the movement traits of the left eye and the right eye were different. Besides, the movement traits of the right eye and left eye were also different in the directions of *X* and *Z*. The fundamental reasons for these differences is that the right eye and the left eye were moved in the reverse direction on the same axis, which means *X* axis, *Y* axis, and *Z* axis. Take an example. The differences of the right eye and left eye on the *Y* axis represents that the left eye is moving downward and the right eye is moving upward, or the left eye is moving upward and the right eye is moving downward. Under that case, the human body’s roll vibration appearance can be decided. Therefore, by means of detecting the movement traits of right eyes and left eyes, whether the human body’s roll vibration appears or not can be decided.

As can be seen from Figure 13, Figure 14, Figure 15, Figure 16, Figure 17, Figure 18, Figure 19, Figure 20, Figure 21, Figure 22, Figure 23, Figure 24 and Figure 25, the experimental results conformed to the actual situation and showed that the eye movements were tracked correctly. This validates the algorithm and indicates that the proposed method is very robust under real conditions. Based on these characteristics, the proposed visual measurement method can be used widely in the future to measure human vibration and evaluate visual fatigue.

## 5. Fatigue Study

**Figure 26:**
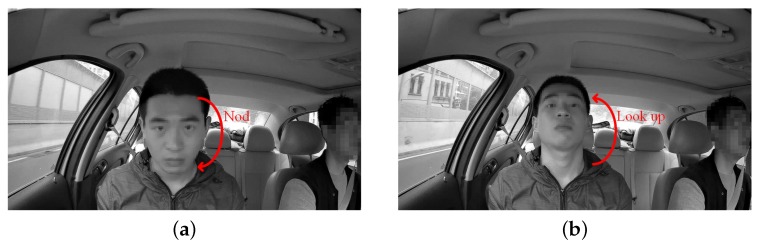
Facial images captured: (**a**) nod; (**b**) look up.

**Figure 27:**
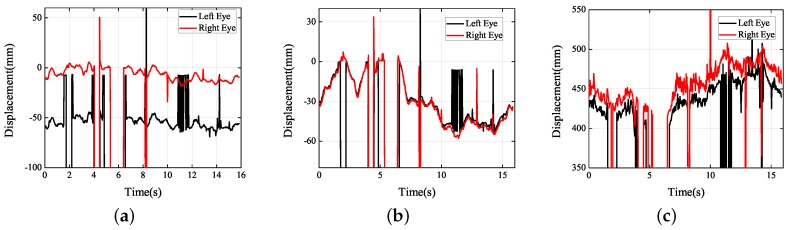
Experimental results obtained (fatigue study): (**a**) *X* axis; (**b**) *Y* axis; (**c**) *Z* axis.

Although the previous part studied the instantaneous visual fatigue caused by acceleration speed and vibration, the driver’s head movement during fatigue driving also needs to be studied to verify that the proposed method can determine the status of the driver’s fatigue. Facial images captured by drive recorder are shown in Figure 26. According to Figure 27b, the subject had two nodding movements at 2–7 s and closed his eyes at 6–7 s. Then, he raised his head, which can be seen from the motion trajectory at 10–15 s. Experiments have shown that when the human eye moves slowly only in the *Y* direction and remains stable for a period of time, the subject is most likely to have a nod or a head-up action. By measuring these small movements, the proposed method can detect whether the driver is tired.

## 6. Limitation

The method proposed in this paper has extremely high stability and can accurately reconstruct the three-dimensional information of the human eye movement in the complex background during the vehicle driving process. However, since the Haar cascade classifier cannot detect closed eyes, the eye movement trajectory cannot be determined for a while when the driver blinks. When the driver takes an extreme posture, the proposed method will assume that the driver is already asleep, resulting in a false alarm. In addition, it takes about 0.5 s to process a 300 × 300 pixel image. This is due to the large amount of calculations performed by the Daugman operator when the motion trajectory is accurately reconstructed. Therefore, before this problem is solved, real-time processing is not possible yet.

## 7. Conclusions

In this paper, we proposed a non-contact videometric measurement method for evaluating the level of fatigue during the vehicle driving process. When performing dynamic calibration, the measurement error was approximately 0.1 mm and the R${}^{2}$ value exceeded 0.9821, which indicates that the driver’s eye trajectories in 3D space can be reconstructed accurately. The proposed method differs from the traditional method of analyzing the driver’s facial expression to determine the driver’s fatigue state. Instead, driver fatigue is evaluated by acceleration and vibration frequency of the human body. The accelerations during car acceleration and braking were 0.03 g and 0.05 g, respectively, while the vibration frequency was 1.3 Hz when car went over speed bumps, from which driver fatigue can be determined. Under real road conditions, braking, speed bumps, and vehicle accelerating experiments were also recorded for a long time. Moreover, appropriate fatigue research was also carried out. In short, the proposed method provides more information about visual fatigue than do two-dimensional detection methods, and it facilitates the design of displays and seats in the future.

## Figures and Tables

**Table 1:** Relationship between acceleration and bodily sensation.

Acceleration	Bodily Sensation
<0.03 g	No sensation
0.03–0.07 g	Very mild discomfort
0.07–0.1 g	Mild discomfort
0.1–0.16 g	Moderate discomfort
0.16–0.25 g	Severe discomfort
>0.25 g	Very severe discomfort

**Table 2:** Fitting evaluation parameters of the reconstruction results.

	Amplitude (mm)	Frequency (Hz)	SSE	R${}^{2}$	RMSE
Ground truth	4.932	4.999	-	-	-
Detection value (L)	5.032	4.999	1.801	0.9994	0.0845
Detection value (R)	4.844	4.999	1.063	0.9996	0.0650

The SSE and RMSE values were all acceptable; the R${}^{2}$ values were close to unity, showing that the fitting accuracy was very high; L and R indicate left eye and right eye, respectively.

**Table 3:** Fitting evaluation parameters of reconstruction results (movement in the *Z* axis).

	Amplitude (mm)	Frequency (Hz)	SSE	R${}^{2}$	RMSE
Ground truth	4.909	4.999	-	-	-
Detection value (L)	4.963	4.999	11.617	0.9821	0.4972
Detection value (R)	4.983	4.999	2.492	0.9966	0.2038

**Table 4:** Acceleration at different speeds.

	Acceleration (Accelerating)	Bodily Sensation	Acceleration (Braking)	Bodily Sensation
10 km/h	-	No sensation	-	No sensation
20 km/h	-	No sensation	0.005 g	No sensation
30 km/h	-	No sensation	0.005 g	No sensation
40 km/h	-	No sensation	0.02 g	No sensation
50 km/h	0.0005 g	No sensation	0.02 g	No sensation

**Table 5:** Amplitude and frequency at different speeds.

	X(mm)	Y(mm)	Z(mm)	Frequency
10 km/h	14	20	30	2
20 km/h	10	10	-	1
30 km/h	5	5	-	2
40 km/h	-	10	30	3
